# A systematic review of the psychometric properties of transition readiness assessment tools in adolescents with chronic disease

**DOI:** 10.1186/1471-2431-14-4

**Published:** 2014-01-09

**Authors:** Lorena F Zhang, Jane SW Ho, Sean E Kennedy

**Affiliations:** 1 Discipline of Paediatrics, School of Women’s & Children’s Health, Medicine UNSW, University of New South Wales, Sydney, Australia; 2 Trapeze Adolescent Service, Sydney Children’s Hospital Network, Sydney, Australia

**Keywords:** Adolescent health, Transition to adult care, Chronic disease, Young adult, Needs assessment

## Abstract

**Background:**

Health care transition of adolescents with chronic conditions may be unsuccessful when patients have not acquired the necessary skills and developmental milestones. It is therefore critical for health care providers to assess the readiness for transition of their adolescent patients. This is currently hindered by the lack of a recognised, well-established transition-readiness assessment tool.

**Methods:**

We conducted a systematic review of all transition-readiness tools for adolescents with chronic medical conditions published in peer-reviewed journals. Tools were rated by the methodological quality of the validation studies, and the psychometric measurement qualities of each tool.

**Results:**

Ten different assessment tools were identified. Seven targeted specific diseases and 3 tools were generic. Most tools were poorly validated with only one tool, the Transition Readiness Assessment Questionnaire (TRAQ) demonstrating adequate content validity, construct validity, and internal consistency.

**Conclusion:**

The TRAQ was the best-validated transition-readiness tool, with additional benefits of disease-neutrality. Further research should focus on testing the predictive validity of this tool, and exploring correlation with transition-outcomes, in an international population.

## Background

Health care transition is the “process of purposeful, planned movement of adolescents with chronic medical conditions from child to adult-centred healthcare systems” [1]. It includes the transition of responsibility from the parent to the child, and preparation for the transfer event [2]. Currently, 90% of adolescents with chronic diseases will survive into adulthood, and will be undergoing this process [3]. Suboptimal transition has detrimental effects on access to medical care, disease outcome, education, and opportunities for a successful adulthood [4].

Transition of adolescents with chronic conditions can be a challenging operation that requires a concerted effort from paediatric and adult health care providers, parents or carers, and individual patients. Much has been written about how to best optimise transition and multiple guidelines have been produced. Most guidelines consistently recommend that paediatric providers should assess an adolescent’s readiness for transition to individualise transition planning. In this regard, the most recent consensus statement from the American Academy of Pediatrics and American College of Physicians [5] states that “practices should select a readiness-assessment tool to use that can be modified for specific patient situations”. The report goes on to state that “regardless of the tool chosen, it should contain specific minimum components that provide an accurate, point-in-time assessment of the individual patient’s ability to transition successfully”. Despite its importance and the availability of a number of tools and checklists, there is evidence to suggest that assessment of readiness for transition is not uniformly performed. For example, a review of 87% of cystic fibrosis (CF) transition programs in the United States found that only 50% perform readiness assessments, <10% have a list of desirable skills, and only 26% of these addressed pertinent skills such as insurance [6]. There is therefore a need to identify a valid, well-established transition readiness tool which can be used in diverse settings.

This systematic review aims to summarise the validation of all published transition-readiness tools for adolescents (aged 11-19 years) with chronic disease. To the best of our knowledge, this is the first review of transition readiness tools, and the authors hope it will clarify which tool is optimal for clinical application.

## Methods

### Search strategy

**Figure 1:**
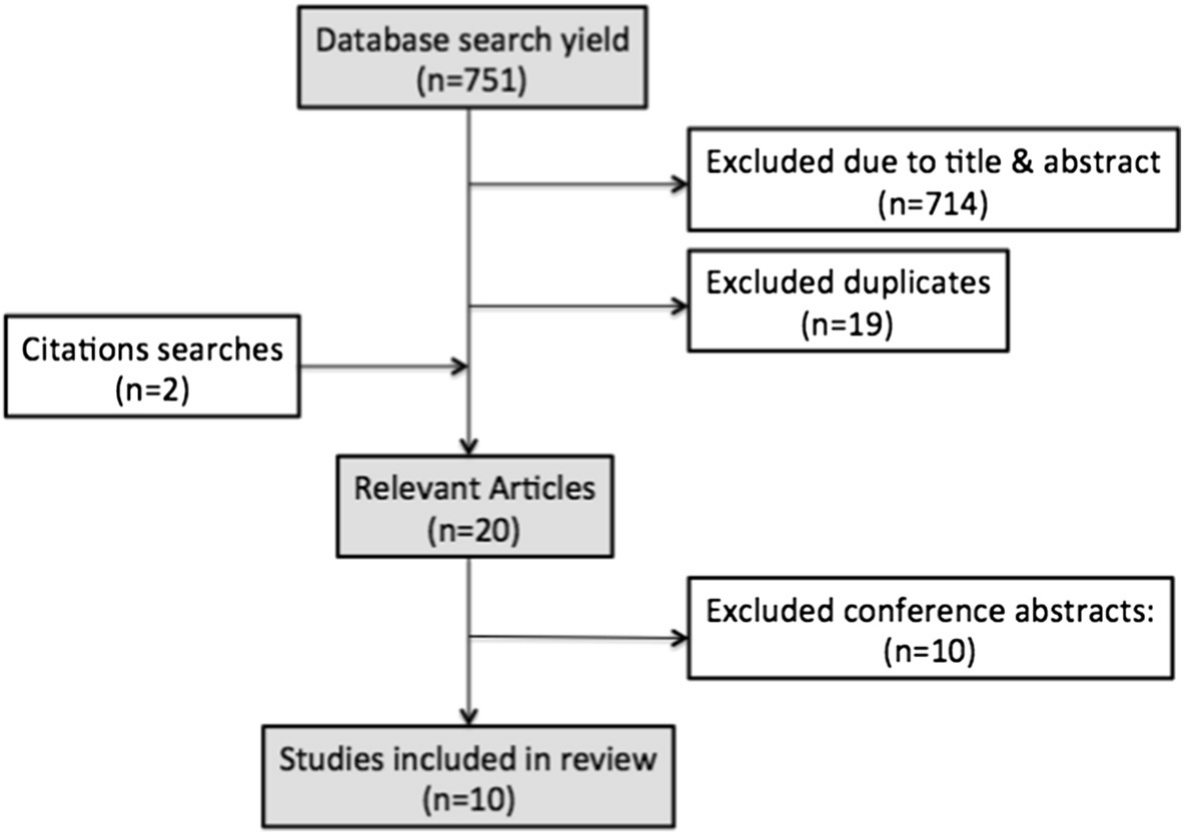
Flow diagram of search strategy.

A literature search of the electronic databases Medline, Web of Science, Embase, CINAHL and PsycInfo, and Google Scholar, was undertaken between February-October 2013. The search terms included permutations of “(transition or transfer) and readiness”, “healthcare (transition or transfer)”, and “adolescents or young adults or children”. We additionally included the terms “assess”, “measure”, “tool”, or “questionnaire”, however these yielded no new results. Citation searches, and reference lists were also reviewed, and the name and primary author of each questionnaire included was searched in Google Scholar for cross-references to the tool. We also searched Google using the phrase “adolescent transition readiness”. Please see Figure 1 for flow diagram of the search, and Additional file 1 for full search strategy.

### Study selection

One reviewer screened each title and abstract for inclusion. All studies which developed, discussed, or evaluated tools for assessing transition readiness involving adolescents (aged 11-19 years) with chronic diseases were included. No restrictions were placed on study design, language, disease or participants. The year of publication was restricted by the databases we searched in: 1806-present (PsycInfo), 1900-present (Web of Science), 1980-present (CINAHL), 1946-present (Medline), and 1947-present (Embase).

### Data extraction

Two independent authors extracted and processed the data, and a consensus agreement was made. Data extracted included characteristics of the design of the tool such as disease-specificity, number of domains, questions, responses, reporters and calculation of scores. Evidence of the applicability of the tool, including cohort demographics, and validity and reliability testing was also extracted.

### Analysis

Assessment of the methodological quality of the validation studies, and the psychometric measurement qualities of the tools was integrated using Terwee’s standardised checklist [7]. Criterion validity was removed from analysis as there is no gold-standard for measuring transition-readiness, and all correlations were with theoretically derived hypotheses (construct validity). More weight was given to content validity, internal consistency, and construct validity when making a quality assessment, as we believe these are the most important properties for a transition-readiness tool.

We also present a descriptive summary of selected studies including our interpretation of the potential utility and limitations of each tool.

## Results

### Search results

Of the 751 results, 20 papers or abstracts on transition-readiness tools were found. Ten validation studies were published in peer-reviewed journals, and these were critically appraised. Ten conference abstracts were also found – 8 of which involved validation of a new tool, and 2 using or re-validating previously validated tools. Numerous checklists were found by searching with Google, including some listed as resources by the American consensus statement [5]. The conference abstracts and general checklists contained inadequate information to allow formal evaluation and were subsequently excluded from analysis.

The tools published in peer-reviewed journals were variable in their design (listed in Table 1) and validation (Table 2). Most tools relied on patient self-report, and included questions on disease knowledge and self-management. Validation by measures of independence, knowledge, or self-management was most common. Seven of the 10 tools were disease-specific, with the majority directed at CF or solid organ transplantation. Most tools scored poorly according to the Terwee criteria (See Table 3) [7].

**Table 1:** Design of transition readiness assessment tools

**TOOL**	**TOOL**						
	**Disease-specificity**	**Number of domains**	**Number of questions**	**What questions are asking?**	**Nature of responses**	**Reporters**	**Calculation of scores**
TRAQ [4]	Chronic diseases	2	33	Skills	5-point Likert scale (Stages of change model)	Patient	1 point each
UNC TRxANSITION [17]	Chronic diseases	10	33	Knowledge & self-management	Interview style (cross-referenced with medical records)	Patient	Each domain=1 Maximum 10
Self-Management Skills Assessment Guide [16]	Chronic diseases	1	21	Health-care awareness & decision-making	5-point Likert scale	Patient & parent	1-5 for each item, total score: 105
SCIS [8]	CF	Multiple	44	Independent knowledge/behaviour	Yes/no	Parent	1point for Yes, 0 for No. Maximum 44
Readiness Questionnaire [9]	CF	2	24	Knowledge & behaviour	Multiple choice or short answer	Patient	1point each, Maximum 24
RTQ [10]	Renal transplant	3	22	Involvement in behaviours & overall transition readiness	4-point Likert scale (not/sometimes/often/always)	Patient & parent	1-4 each question Maximum 48
TRS [2]	Liver transplant	4	Patient: 42. Parent: 36	Skills, knowledge, behaviour	Likert scale & skill demonstration	Patient & parent	Items vary, Maximum 126 (parent: 108)
RCBRS [13]	T1DM	1	7	Readiness to change responsibility	5-point scale (Stages of change model)	Patient & parent	1-5 each item, maximum 35
McPherson et al. [14]	Sickle cell disease	5	NR	NR	Variable, mostly 3-point Likert Scale (knowledge section worth 4 points)	Patient	NR (high score = more ready for transfer)
TRQ [15]	HIV	6	21	Knowledge of disease & transition process	NR	Patient	1point each, 2 for disease knowledge

CF, cystic fibrosis; T1DM, type 1 diabetes mellitus; HIV, human immunodeficiency virus; NR, not reported.

**Table 2:** Cohort characteristics of transition readiness assessment tools

**TOOL**	**COHORT CHARACTERISTICS**					
	**Disease**	**Number**	**Age (years)**	**Time since diagnosis/transplant**	**Race & gender**	**Country of validation**
TRAQ [4]	Chronic diseases	192	16-26 (mean: 19.7)	NR	64% white, 56% female	USA
UNC TRxANSITION [17]	Chronic diseases	NR	12-22	NR	NR	USA
Self-management skills assessment guide [16]	Chronic diseases	49	11-19 (mean: 15.6)	NR	86% Caucasian	Canada
SCIS [8]	CF (with pancreatic insufficiency)	76 patients, 70 parents	4-17 (mean: 11.2)	NR	94% white	USA
Readiness questionnaire [9]	CF	36	NR	NR	NR	Canada
RTQ [10]	Renal transplant	48 patients, 32 parents	15-21 (mean: 18.6)	5.73 y	58% white, 29% black, 10% Hispanic	USA
TRS [2]	Liver transplant	71 patients, 58 parents	11-20 (mean: 15.6)	1-19 y (mean: 9.4 y)	56% female	USA
RCBRS [13]	T1DM	69	12-17 (mean: 14.2)	>6 months (mean: 5.49)	87% Caucasian	USA
McPherson et al. [14]	Sickle cell disease	69	14-21	NR	43% female	USA
TRQ [15]	HIV	65	9-25 (mean: 15.8)	1-17 years (mean: 10.5)	44% female, 45% Caucasian	USA

CF, cystic fibrosis T1DM, type 1 diabetes mellitus; HIV, human immunodeficiency virus; NR, not reported.

**Table 3:** Scoring of psychometric measures of transition-readiness tools by Terwee criteria

**Tool**	**Content validity**	**Internal consistency**		**Construct validity**	**Reproducibility**		**Responsiveness**	**Floor & ceiling effects**	**Interpretability**
		**Factor analysis**	**Cronbach’s alpha**		**Agreement**	**Reliability**			
TRAQ [4]	+	+FA	+: total (0.93), domain 1 (0.92), domain 2 (0.82)	+: 100% (age, gender, disease type)	0	0	0	0	? + mean/SD 0 MIC
		?sample size							
UNC TRxANSITION [17]	+	0	0-? used PC	0: inferred from development	+	? Small cohort (n = 35)	0	0	0
Self-management skills assessment guide [16]	–	0	+: 0.93	+: 100% (correlation with independence)	0	? inter-rater PC 0.56 Small cohort (n = 47)	0	+	0
SCIS [8]	-	0	+: 0.93	?: no hypotheses, correlates with age, years since diagnosis	?: Small cohort (n = 35)	0	0	0	0
Readiness auestionnaire [9]	-	0	0	+: 100% (caregiver ratings)	0	? inter-rater PC 0.65 Small cohort (n = 36)	0	0	0
RTQ [10]	-	0	+: 0.79-0.94	+: 86% (responsibility, medication knowledge, self-refilling, family relationship, decreased family involvement, adherence), no correlation with age	0	? inter-rater PC 0.5-0.68 Small cohort (n = 48)	0	0	0
TRS [2]	-	+FA ?sample size	-: 0.19-0.85	-: 50% (self-management, age NOT adherence or health outcomes)	0	0	0	+	? ? mean/SD (age) 0 MIC
RCBRS [13]	-	0	+: 0.85-0.9	+: 100% (responsibility, self-efficacy, decreased parenting stress)	0	? inter-rater PC 0.33-0.58	0	0	? + mean/SD ? MIC
McPherson et al. [14]	0	0	0	+: 100% (age, disease severity, gender)	0	0	0	0	? + mean/SC ? MIC
TRQ [15]	-	0	0	+: 80% (anxiety, confidence in GP, decreased treatment length, improved with intervention)	0	0	0	0	0

FA: Factor analysis, K: weighted-kappa, PC: Pearson’s correlations, MIC: minimal important change.

### Disease specific tools

The Self-Care Independence Scale (SCIS) is a 44-item carer-report questionnaire assessing the child’s ability in and knowledge of, disease management. It was tested on 75 families who had 4-17 year olds suffering from CF with pancreatic insufficiency [8]. The majority of children were Caucasian, of above average intelligence and above average socioeconomic status. This scale did not receive a positive rating for any measure in the Terwee criteria. A factor analysis of the scale wasn’t reported and thus its internal consistency is indeterminate despite an excellent Cronbach’s alpha (α = 0.93). The reproducibility of the scale appeared good due to a test-retest correlation of (r = 0.81), however the sample size of 35 did not meet the minimal criteria of 50 proposed by Terwee [7]. The scale correlated with age (r = 0.67), years since diagnosis (r = 0.58), CF knowledge (r = 0.62), and general independence as determined by the 21-item validated Highland Dependency Questionnaire (r = 0.62), however the authors did not report their hypothesis and thus the construct validity is indeterminate. It also has a very specific cohort and consequently, the validity of the tool in other patient groups is uncertain [8].

Cappelli *et al.* developed another CF-specific questionnaire with 21 items testing disease knowledge and behaviour [9]. It was validated in Canada by comparing the total readiness score with nominal caregiver ratings of either able or not able to cope with transfer. According to this measure, 77% of the adolescent respondents were correctly classified by summated questionnaire scores. Some limitations of this study are that content validation did not involve input from adolescents, and reliability was largely untested.

The Readiness for Transition Questionnaire (RTQ) is a 10-item tool for patients with kidney transplants. Notably, it uses multiple reporters, has an additional question about ‘overall transition readiness’, and also includes an assessment of non-adherence [10]. Non-adherence is a significant barrier to successful transition as it is thought to be the cause of the high rates of kidney transplant failure in adolescents and young adults [10]. The construct validity of the RTQ was therefore assessed using the Medical Adherence Measure. Medication adherence was found to contribute 33% of the variance of overall transition readiness scores, suggesting that adherence is a strong indicator of transition-readiness. However, despite transition readiness scores increasing with age, adherence actually decreased, raising the possibility that non-adherence occurs independently of other aspects of transition readiness. The RTQ also correlated with adolescent responsibility (r = 0.68), decreased parental involvement (r = -0.39), medication knowledge, self-refilling behaviour, and family relationship. There was a good Cronbach’s alpha, however internal consistency was rated as indeterminate due to a lack of factor analysis. Reliability was additionally scored as indeterminate because inter-rater reliability was tested using Pearson’s correlations (r = 0.5-0.68) without a weighted kappa.

Adherence, measured by blood levels of immunosupressants, was also used to validate the Transition Readiness Scale (TRS) for patients with liver transplants [2]. The authors reported psychometrics for both an adolescent and parent version. A factor analysis was performed, however there was wide variability in Cronbach’s alphas. Most domains had good Cronbach’s alphas, but some scored <0.7. Construct validity was questionable as adherence and health outcomes did not correlate with total score. It should be noted that measuring non-adherence is a difficult and inexact science and although frequently used, both patient self-report and therapeutic drug monitoring may underestimate the extent of non-adherence [11,12].

Kaugars *et al.* developed a 7-item questionnaire to assess the readiness of patients with Type 1 Diabetes, and their parents, to change the balance of responsibility of disease management [13]. It was validated on 69 families in the US by correlation with self-efficacy scores (r = 0.90), decreased parental stress (r = 0.94), and responsibility. It has good Cronbach’s alpha (α = 0.85-0.9), but factor analysis and weighted kappa were not reported. There was poor inter-rater correlation between parent and patient (r = 0.58) and between mother and father (r = 0.33). Furthermore, it only assesses readiness to change responsibility and does not assess transition-readiness directly. Similar to the SCIS [8], the study cohort was quite homogenous being 87% Caucasian, and 90% of parents having a college education.

Mcpherson *et al.* employed a sickle cell disease-specific questionnaire with 5 domains: knowledge, thought, interest, difficulty, and importance of transition. Interest and knowledge domains correlated with age and disease severity in a group of 70 adolescents from a single centre. Reliability, reproducibility, and content validity were not reported, and there was a high non-response rate of 71% [14].

The Transition Readiness Questionnaire (TRQ) is a 21-item HIV-specific questionnaire that was administered to patients before and after attending a transition program, an average of 6.8 months apart. The questionnaire assesses six variables including ability to arrange appointments, awareness of financial factors and knowledge of disease status and medications. Construct validity was adequate; transition-readiness scores were found to improve with time and were inversely related to state anxiety at baseline. However reliability and reproducibility testing were not reported [15]. Although there is more focus on the improvement of scores with transition programs than the accuracy of the tool in predicting transition-readiness, this study highlights a purpose of the tool in identifying ‘problem areas’ which can then be targeted by transition programs.

### Disease-neutral tools

The Self-management Skills Assessment guide is a 21-item youth and parent questionnaire. The scores increased with general independence as assessed by the Highland Questionnaire. Scores did not correlate with age, gender, ethnicity, or parent education. Internal consistency is indeterminate despite good Cronbach’s alphas (α = 0.89-0.93) as factor analysis was not reported [16]. As with the SCIS, it measures self-management skills as a construct of transition-readiness, and although this relationship is theoretically assumed, evidence supporting it is minimal.

The Transition Readiness Assessment Questionnaire (TRAQ) involves 33 questions assessing skills/actions from 2 domains: self-management and self-advocacy, with 5 responses adapted from the ‘Stages of Change’ model. It was validated on 192 patients at 2 sites where the TRAQ score, as hypothesised, correlated with age, gender, and disease groups, but not race. It has excellent internal consistency with a Cronbach’s alpha of 0.93 and was one of two studies which conducted a factor analysis, although the sample size was arguably too small (6 patients/item, as opposed to 7 recommended by Terwee [7]). It was also one of two tools whose development included a pilot on a group of adolescents, and thus received a positive rating for content validity. Its test-retest and inter-rater reliability were not reported [4].

Ferris *et al.* suggested that the TRAQ’s validity may be impaired as it relies on self-reporting, and instead offered the UNC TR[x]ANSITION, a tool with 33 questions across 10 domains which can be cross-referenced with medical records [17]. It was the only other study that received a positive score for content validity, however construct validity wasn’t analysed and instead inferred from the development of the tool (interview of transition experts and 3 pilot tests on 185 adolescents in total). The authors did not report internal consistency by factor analysis or Cronbach’s alphas, and the inter-rater reliability (κ = 0.71) was performed on a relatively small cohort.

The only study of transition-readiness to originate outside of North America was a large exploratory study of factors that contribute to transition-readiness in Dutch adolescents [18]. No transition-readiness assessment tool was developed, and instead, item specific scores were compared to each participant’s self-assessment of transition-readiness. Eleven variables significantly contributed to transition-readiness, including demographic factors (age, gender, ethnicity), attitude towards transition, impact of the disease, and health care self-efficacy. The main limitation of the study is the reliance on self-report for transition-readiness. The non-response rate was also notably high (64%).

## Discussion

### Principal findings

This review shows that the psychometric properties of available transition readiness tools are limited or untested. None of the tools received positive ratings in the most important measurement properties: content validity, internal consistency, and construct validity. The TRAQ was evaluated as the best tool as it had positive scores for content and construct validity, and included a factor analysis.

The systematic review found 2 types of tools: those which are aimed at a specific disease type, and those which are aimed at chronic disease in general. A disease-specific tool negates criticism of self-report by testing disease-specific knowledge (e.g. “demonstrate how you would use an inhaler”) [17], however as transition issues are common to all adolescents with chronic diseases [18], a transition-readiness tool applicable to multiple diseases would offer several advantages. A disease-neutral tool enables assessment of less common diseases where tools haven’t been developed, allows larger sample sizes, and focuses research on a single tool.

The phrasing of questions in the UNC TR[x]ANSITION tool (e.g. “what medications are you taking” and “explain how you take these”) allows cross-referencing of patient responses with medical records, and overcomes the disadvantage of self-report [17]. Notable features of other tools include the use of multiple reporters to improve validity (RTQ, TRS, RCBRS), the inclusion of an ‘overall transition readiness’ question (RTQ), and the measurement of adherence due to its impacts on disease outcome and thus transition decisions. Useful domains include involvement in skills/behaviours, disease knowledge, and transition knowledge.

### Limitations of existing studies

The criterion validity of transition-readiness tools is difficult to establish when there is no ‘gold standard’ measure of transition readiness. Most of the tools have been validated by measures of self-efficacy, medication knowledge, or age (which is known to be a poor measure of transition readiness). As much of the value of a transition-readiness tool is in its ability to time transition for optimal health outcomes, what is necessary is a longitudinal study of the tool’s ability to predict future transition outcomes. These outcomes could be disease-neutral (e.g. number of hospital admissions), or disease state-specific (e.g. organ function tests, number of rejection episodes).

Most of the studies evaluated also excluded patients with cognitive impairment. Many adolescents with chronic diseases also have general cognitive impairment or selective learning problems and these patients may need the most assistance with transition. A disease-neutral tool focusing on self-report may not be practical in this group, and they may benefit more from a disease-specific tool which can cater to their needs (e.g. via parent report). Further validation studies need to be conducted on these groups and in different language groups, or other tools developed which suit their needs.

Currently, all the validation studies originate from USA or Canada. The validity of specific content or overall scores needs to be tested in culturally diverse areas and in different health care settings. One difference in health care provision between nations is the ability of paediatric clinicians to continue to care for young adults. For example, in the United Kingdom and Australia, the licensing and funding arrangements are such that children’s hospitals do not admit patients older than 16-18 years. Interestingly, one conference abstract found in the search described a Canadian validation study of the TRAQ which found that the TRAQ was not valid in younger patients with a mean age of 15.3 years [19]. This raises questions about the validity of these tools in a country with a different healthcare system and different patient mix, and supports the need for on-going validation trials.

It is worth noting that the literature search uncovered many abstracts of recent conference presentations that included studies of new transition readiness tools, suggesting that this is a vibrant and growing area of research and clinical practice. It should also be acknowledged that our results are based solely on psychometric studies found in the peer-reviewed literature. It is possible that other tools and checklists have been validated, we did not contact the authors of tools found in the web search.

## Conclusion

There have been recent advances in the development of a transition readiness tool, however most of these require further validation before they can be broadly recommended for clinical practice. Although disease-specific tools predominate, disease-neutral tools have additional advantages for research and clinical application, and focus should be placed on conducting a longitudinal study of a transition tool such as the TRAQ in predicting health outcomes. A reliable and valid transition readiness tool may dissipate some of the uncertainty around the transition process and allow for tailoring of programs to suit the patients’ transition needs.

## Abbreviations

CF: Cystic fibrosis; SCIS: Self-care independent scale; TRQ: Transition readiness questionnaire; HIV: Human immunodeficiency virus; TRAQ: Transition readiness assessment questionnaire; RTQ: Readiness for transition questionnaire; TRS: Transition readiness scale; RCBRS: Readiness to change the balance of responsibility scale; T1DM: Type 1 diabetes mellitus; NR: Not reported; FA: Factor analysis; K: Weighted-kappa; PC: Pearson’s correlations; MIC: Minimal important change.

## Competing interests

The authors declare that they have no competing interests.

## Author’s contributions

LZ and SK developed the study design and carried out the systematic review. LZ was responsible for the data analyses, preparing the initial manuscript, and subsequent redrafting. SK provided a significant level of guidance on the review design including data analysis, and was involved in redrafting. JH advised on study design and contributed to manuscript preparation. All study authors approved the final version of the manuscript.

## Pre-publication history

The pre-publication history for this paper can be accessed here:

http://www.biomedcentral.com/1471-2431/14/4/prepub

## Supplementary Material

Additional file 1 Full search strategy in pdf. Click here for file
