# Supplementary figures and images for: A systematic review of the psychometric properties of transition readiness assessment tools in adolescents with chronic disease
Source: BMC Pediatr. 2014 Jan 9;14:4. doi: 10.1186/1471-2431-14-4 (PMC3898257; doi:10.1186/1471-2431-14-4)

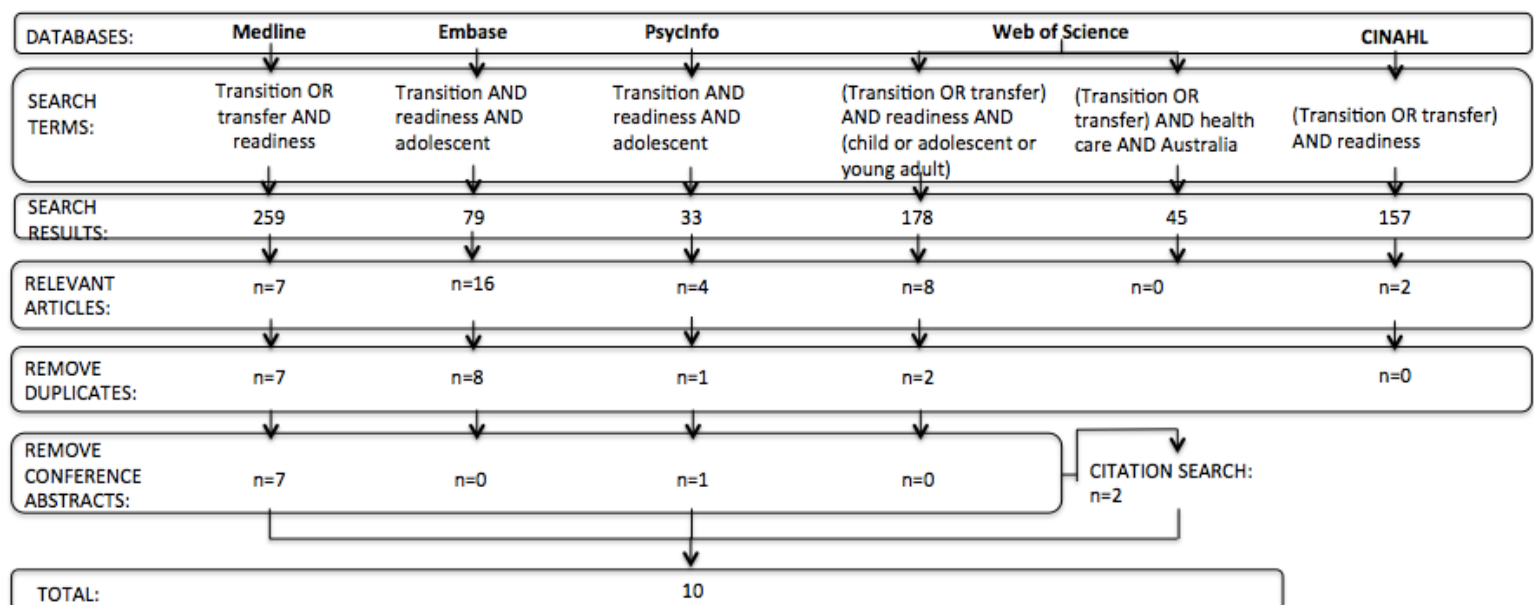

Supplement: Additional file 1 — Full search strategy in pdf. [file 1471-2431-14-4-S1.pdf]
